# Determination of the efficacy and side-effect profile of lower doses of intrathecal morphine in patients undergoing total knee arthroplasty

**DOI:** 10.1186/1471-2253-8-5

**Published:** 2008-09-24

**Authors:** Patrick Hassett, Bilal Ansari, Pachaimuthu Gnanamoorthy, Brian Kinirons, John G Laffey

**Affiliations:** 1 Department of Anaesthesia, Galway University Hospitals and National University of Ireland, Galway, Ireland; 2 Centre for Pain Research, National University of Ireland, Galway, Ireland

## Abstract

**Background:**

Intrathecal (IT) morphine provides excellent post-operative analgesia, but causes multiple side effects including nausea and vomiting (PONV), pruritus and respiratory depression, particularly at higher doses. The lowest effective dose of spinal morphine in patients undergoing total knee arthroplasty is not known.

**Methods:**

We evaluated the analgesic efficacy and side effect profile of 100 – 300 μg IT morphine in patients undergoing elective total knee replacement in this prospective, randomized, controlled, double-blind study. Sixty patients over the age of 60 undergoing elective knee arthroplasty were enrolled. Patients were randomized to receive spinal anaesthesia with 15 mg Bupivacaine and IT morphine in three groups: (i) 100 μg; (ii) 200 μg; and (iii) 300 μg.

**Results:**

Both 200 μg and 300 μg IT morphine provided comparable levels of postoperative analgesia. However, patients that received 100 μg had greater pain postoperatively, with higher pain scores and a greater requirement for supplemental morphine. There were no differences between groups with regard to PONV, pruritus, sedation, respiratory depression or urinary retention.

**Conclusion:**

Both 200 μg and 300 μg provided comparable postoperative analgesia, which was superior to that provided by 100 μg IT morphine in patients undergoing total knee arthroplasty. Based on these findings, we recommend that 200 μg IT morphine be used in these patients.

**Trial registration:**

ClinicalTrials.gov Identifier NCT00695045

## Background

The provision of high quality postoperative analgesia after total knee arthroplasty continues to present a challenge. Systemic opioids can cause post operative nausea and vomiting, sedation, and respiratory depression, which may be especially undesirable in older patients with co-existing respiratory disease, such as patients undergoing major joint replacement surgery. Intrathecal (IT) opiates are a useful option in patients undergoing knee arthroplasty, and can markedly reduce postoperative opioid requirements [1-3].

However, the use of IT morphine may be associated with a number of distressing side effects e.g. pruritus, urinary retention, nausea and vomiting [3-7], and potentially life-threatening adverse effects i.e. delayed respiratory depression [2,3,8,9]. Postoperative nausea and vomiting (PONV) following IT morphine may prove particularly difficult to control [10], perhaps as a result of the potential for IT morphine to delay gastric emptying [5]. The hydrophilic properties of morphine contribute to both the longevity of its analgesic action and also the risk of late respiratory depression due to rostral spread when administered intrathecally. Profound late respiratory depression has been reported in a number of earlier studies, albeit following larger doses of spinal morphine than is used in current practice [11].

The efficacy of doses of IT Morphine of 300 μg or greater, in patients undergoing total knee arthroplasty, has been well demonstrated [2,3]. However, the incidence of PONV is high, with an incidence of 60% reported [10]. Of greater concern is the potential for respiratory depression, even with doses as low as 300 μg of IT Morphine [2,12]. The potential for lower doses of IT Morphine to provide effective analgesia, while reducing postoperative side-effects, remains unclear. One recent study demonstrated that 500 μg intrathecal morphine produced better analgesia than 200 μg after knee replacement [1]. Rathmell et al could not find evidence that doses of IT morphine up to 300 μg provided significant analgesia in this population [13]. Conversely, Cole et al demonstrated that 300 μg IT morphine did produce effective analgesia following total knee arthroplasty [2].

The aim of this study was to determine the analgesic efficacy and side-effects profile of 100 μg and 200 μg of IT morphine, in comparison to 300 μg, in patients undergoing elective total knee arthroplasty.

## Methods

After obtaining approval by the Hospital Ethics Committee, and written informed patient consent, we enrolled ASA physical status I-III patients scheduled for elective total knee arthroplasty in a prospective, randomized, double blind, controlled clinical trial. Exclusion criteria included non-suitability for spinal anaesthesia as deemed by the anaesthetist providing care for the patient; history of allergy or severe nausea with opioid analgesics or local anesthetic agents; or any medical condition resulting in ASA status greater than III. All patients received spinal anesthesia. The allocation sequence was generated by random number tables, and the allocation concealed in sealed envelopes, which were not opened until patient consent had been obtained. Patients were randomized to receive one of three IT doses of preservative free morphine: 100 μg, 200 μg, and 300 μg.

No patient received premedication. Spinal anesthesia was performed with the patient in the sitting position at the L3/4 intervertebral space utilizing a 25 gauge Whitacre needle. Patients were administered 15 mg of hyperbaric bupivacaine (Marcain Heavy^®^, AstraZeneca Group, Luton, UK), and either 100 μg, 200 μg or 300 μg of preservative free morphine into the intrathecal space, depending on group allocation. A total volume of 4 ml was administered to each patient, with the balance of the IT injectate composed of 0.9% NaCl. The dose of IT morphine administered was not noted in the patients anesthesia record; however this was documented in a sealed envelope attached to the patients anesthesia record which could be opened in case of an adverse event. All patients received 40% oxygen via face mask for the duration of the procedure. Standard monitoring, including non-invasive blood pressure, electrocardiogram, and oxygen saturations were used in all patients for the duration of surgery.

At the end of the procedure, all patients received 100 mg of diclofenac sodium per rectum, and were then transferred to a High Dependency Unit (HDU) for a period of 24 hours post-operatively. All patients received nurse administered 2 mg intravenous morphine bolus on demand. Each administration was initiated by patient request and a minimal interval of ten minutes was required between of each morphine bolus. First line treatment of PONV consisted of prochlorperazine 12.5 mg by intramuscular injection. If this proved ineffective second line therapy consisted of 4 mg of ondansetron by intravenous injection. Each administration was initiated by patient request and a minimal interval of one hour was required between administration of first and second line agents. Treatment for pruritus consisted of promethazine 20 mg by intramuscular injection six hourly and treatment in all cases was initiated upon patient request. Naloxone was reserved for treatment of pruritus that was resistant to promethazine therapy.

Patients were monitored for 24 hours by the HDU nursing staff, who were unaware of the patient allocation. Assessment parameters included severity of postoperative pain, and the presence and severity of postoperative nausea and vomiting (PONV), pruritus, sedation, and respiratory depression. The primary outcome variable, the severity of pain in the first 24 h postoperatively, was assessed every 4 hours utilizing a Visual Analogue Scale (VAS) and by the amount of supplemental morphine required in the first 24 hours. In addition, the time to first request for supplemental analgesics was recorded.

The presence and severity of PONV was assessed every 4 hours using an ordinal scale [0 = no nausea; 1 = mild; 2 = moderate; 3 = severe; 4 = Vomiting]. In addition, the time to first request for antiemetic therapy, and total amount of supplemental antiemetic administered, was recorded. The presence and severity of pruritus was assessed every 4 hours using an ordinal scale [0 = no itch; 1 = mild; 2 = moderate; 3 = severe]. The time to first request for therapy for pruritus, and total amount of promethazine administered, were recorded.

Respiratory rate and arterial oxygen saturation were assessed on a continuous basis over the 24 hour post-operative period. The incidence of (i) reduced respiratory rate, defined as respiratory rate < 12 breaths per minute; and (ii) mild (SaO_2 _< 94% on room air) moderate (SaO_2 _< 90% on room air) and severe (SaO_2 _< 85% on room air) arterial hypoxemia were recorded. At each 4 hourly data collection point, the lowest respiratory rate and lowest arterial oxygen saturation in the previous 4 hours were recorded.

Sedation was scored according to the following scale (1 = alert; 2 = calm; 3 = drowsy; 4 = sleeping, easily arousable; 5 = sleeping, difficult to arouse) and the need for urethral catheterization (defined as absence of spontaneous voiding 8 h after surgery and urine volume at catheterization of >400 ml) was recorded.

### Statistical analysis

We based our sample size estimation on the postoperative morphine requirement. We considered that a clinically important reduction would be a one third decrease in postoperative morphine requirements. Based on pilot studies we projected this reduction to be 5 mg, with a projected standard deviation of 5 mg. Based on these figures, using an α = 0.05 and β= 0.2, for an experimental design incorporating three equal sized groups, we estimated that 20 patients would be required per group. We therefore aimed to enroll 60 patients into this study.

All analyses were performed on an intention-to-treat basis. The analysis was preformed using Sigmastat 3.5 (Systat Software, San Jose, CA, USA). Demographic data were analyzed using one way analysis of variance (ANOVA), ANOVA on ranks, or χ^2 ^analysis where applicable. For VAS data, group comparisons were performed at each time point using one way ANOVA with post hoc comparisons using Student-Newman-Keuls. Area under the VAS-time curve (AUC) was calculated by the trapezoidal method for each patient and compared using one way ANOVA. Ordinal data were analyzed using ANOVA on ranks with post hoc comparisons performed using Mann Whitney U test with the Bonferroni correction for multiple comparisons. The incidence of side-effects such as pruritus was compared using the Chi squared test for multiple variables.

Continuous data are presented as means ± standard error of the mean (SEM), ordinal data are presented as medians ± quartiles (interquartile range), and categorical data are presented as raw data or as frequencies. The α level for all analyses was set as P < 0.05.

## Results

Eighty patients were assessed for eligibility to participate in the study [See Additional file 1]. Of these 80 patients, 61 fulfilled eligibility criteria and were asked to participate. One patient refused consent, and the remaining 60 were included in the study. The 60 patients recruited to participate in the study were equally distributed between the groups and there were no between group demographic differences (Table 1). All patients randomized to treatment allocation completed the study, and their data was included in the analysis. Of the 19 patients that were not eligible for inclusion, 15 had been deemed by their anaesthetist not to be suitable for spinal anaesthesia, while 4 patients had a history of opioid induced nausea and vomiting.

**Table 1:** Demographic data and perioperative factors.

**Variable**	**100 μg IT Morphine**	**200 μg IT Morphine**	**300 μg IT Morphine**
Number of Patients	20	20	20
Age (yr)	69.2 ± 1.6	70.6 ± 2.0	73.9 ± 1.3
Weight (kg)	84.0 ± 2.3	75.7 ± 2.8	86.2 ± 3.4
Gender (m/f)	8/12	8/12	11/9
Duration of Procedure (mins)	101.3 ± 9.0	94.5 ± 7.4	95.8 ± 7.6
24 hr Blood Loss (mL)	518.0 ± 22.7	498.0 ± 27.6	526.5 ± 29.5
Fluids Administered in 24 hrs (Litres)	5.2 ± 0.2	5.3 ± 0.2	5.6 ± 0.2

Data represented as Mean +/- SEM.

Abbreviations: IT, intrathecal.

**Figure 1:**
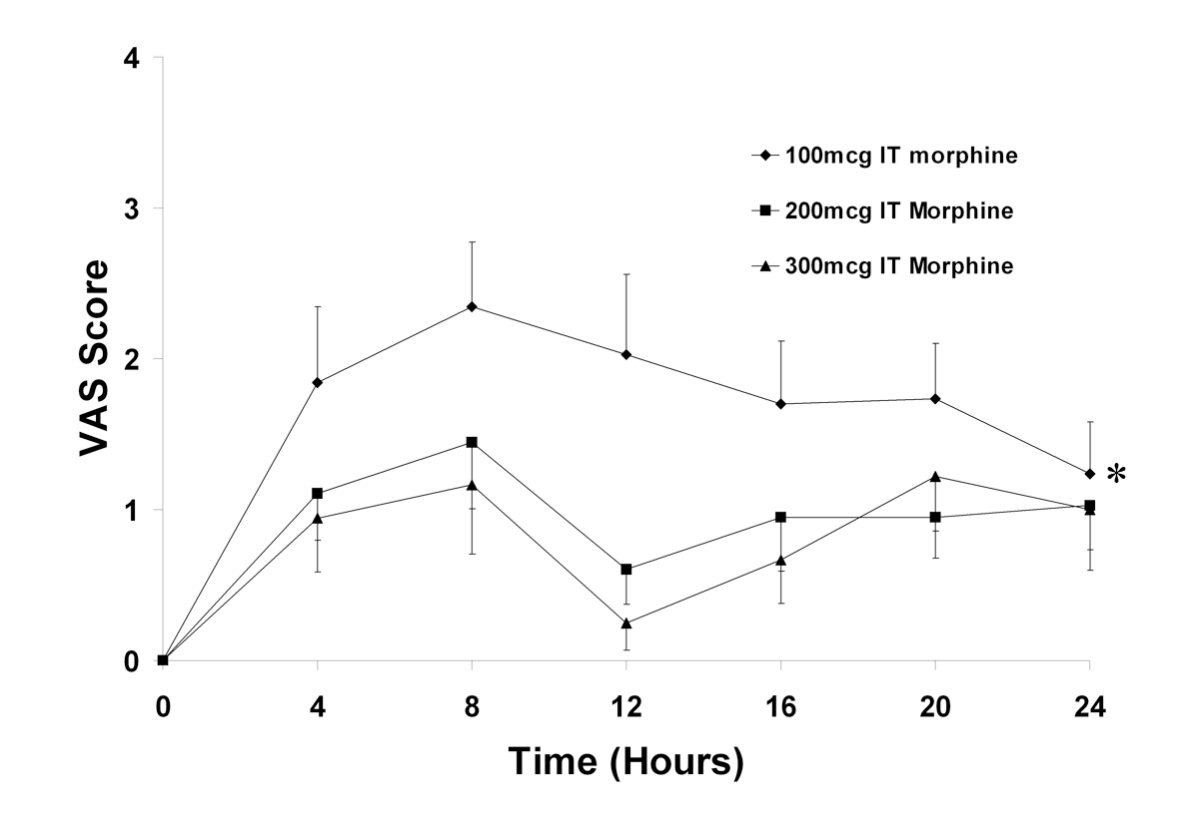
**Mean (+/- SEM) postoperative visual analogue scale (VAS) pain score in each group, measured every 4 hours, for the first 24 postoperative hours**. * Indicates significantly higher Area under VAS-Time curve compared to the 200 μg and 300 μg IT Morphine groups (P = 0.006, one way ANOVA).

**Figure 2:**
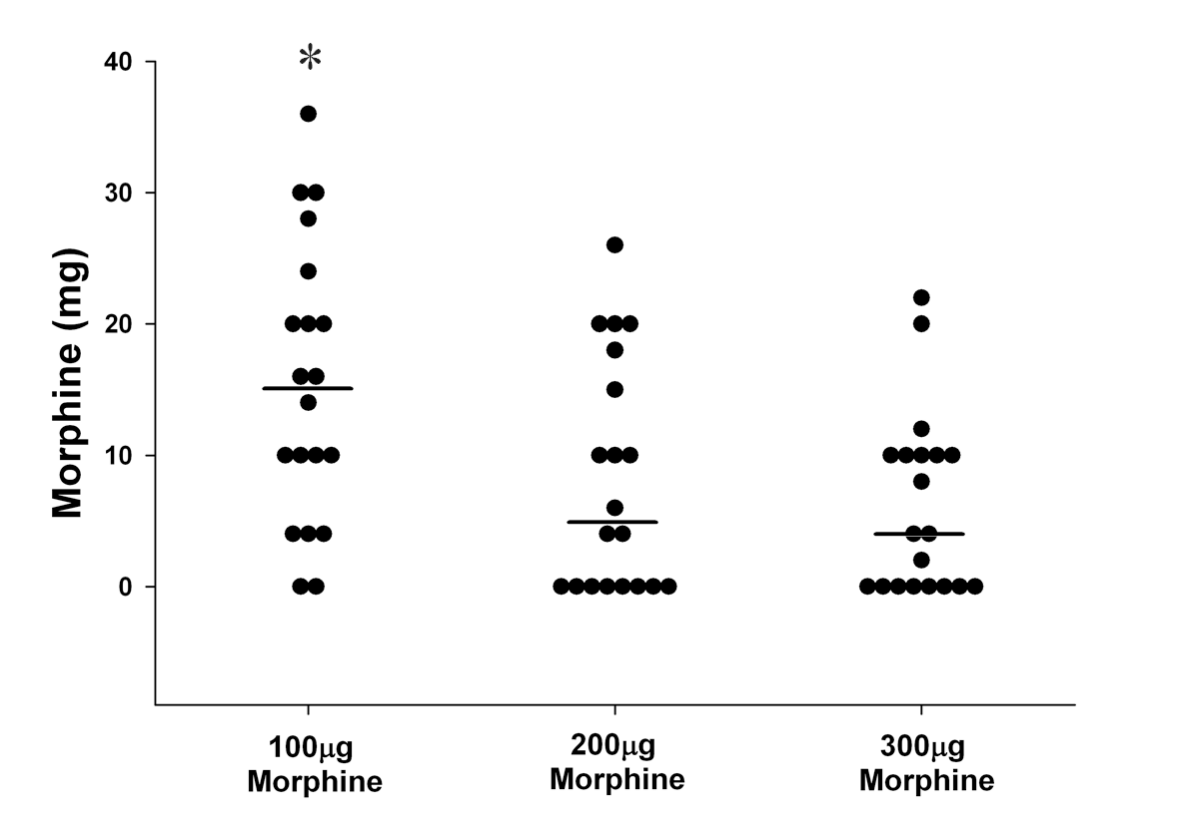
**Dot plot of postoperative requirement for supplemental morphine over the first 24 postoperative hours for each patient in each group. The middle line for each group represents the median value**. * Indicates significantly higher morphine requirements compared to the 200 μg and 300 μg IT Morphine groups (P = 0.001, Kruskall-Wallis one way ANOVA on ranks).

Both 200 μg and 300 μg of IT morphine provided comparable analgesia, in terms of postoperative VAS scores (Figure 1), median amount of supplemental morphine required (Figure 2), and time to first request for supplemental analgesia (Table 2). In contrast, patients that received 100 μg had lower quality analgesia compared to the patients that received 200 μg or 300 μg of IT Morphine. The area under the VAS-time curve was significantly greater for the patients that received 100 μg (41.1 ± 6.7) compared to the patients that received 200 μg (22.2 ± 3.8) or 300 μg (18.6 ± 4.2) of IT Morphine (P = 0.006, One way ANOVA) (Figure 1). Postoperative pain scores were higher in the group that received 100 μg compared to the patients that received 200 μg or 300 μg of IT Morphine (Figure 1). Median supplemental morphine requirements were significantly lower in the groups that received 200 μg and 300 μg compared to that which received 100 μg of IT morphine (Figure 2).

**Table 2:** Data regarding requirement for postoperative analgesic, anti-emetic and anti-pruritic therapy and postoperative respiratory function.

**Variable**	**100 μg IT Morphine**	**200 μg IT Morphine**	**300 μg IT Morphine**	**P Value**
Median time (mins) to first request for Rescue Analgesia (interquartile range)	442 (240, 1100)	607 (343, 1440)	857 (397, 1440)	0.2
Number of patients that requested Rescue Analgesia	17/20	12/20	12/20	0.1
Number of patients that requested anti-emetic therapy	7/20	9/20	11/20	0.4
Time to first request for Antiemetic Therapy (mins)	1117 ± 116	1037 ± 109	809 ± 138	0.2
Number of patients that required anti-pruritic therapy	0/20	3/20	3/20	0.2
Incidence of Respiratory Depression (Total episodes of Resp Rate < 12)	3/20	5/20	5/20	0.7
Number of episodes of mild arterial hypoxemia (SpO_2 _90 – 94%)	8/20	9/20	8/20	0.9
Number of episodes of moderate arterial hypoxemia (SpO_2 _85 – 90%)	1/20	0/20	1/20	0.6
Number of episodes of severe arterial hypoxemia (SpO_2 _< 85%)	0/20	0/20	1/20	0.4
Median Sedation Score (interquartile range)	1 (1, 4)	1 (1, 4)	1 (1, 4)	0.9
Number of episodes of significant sedation (Total episodes of Sedation score = 5)	4/20	3/20	4/20	0.9

**Notes**: Data are presented as number of patients [from a maximum of 20] for each category unless otherwise stated. Data for time to request antiemetic therapy is presented as time in minutes +/- SEM. Sedation scores are presented as medians and interquartile ranges.

Abbreviations: IT, intrathecal; resp, respiratory; SpO_2_, arterial haemoglobin oxygen saturation; mins, minutes

**Figure 3:**
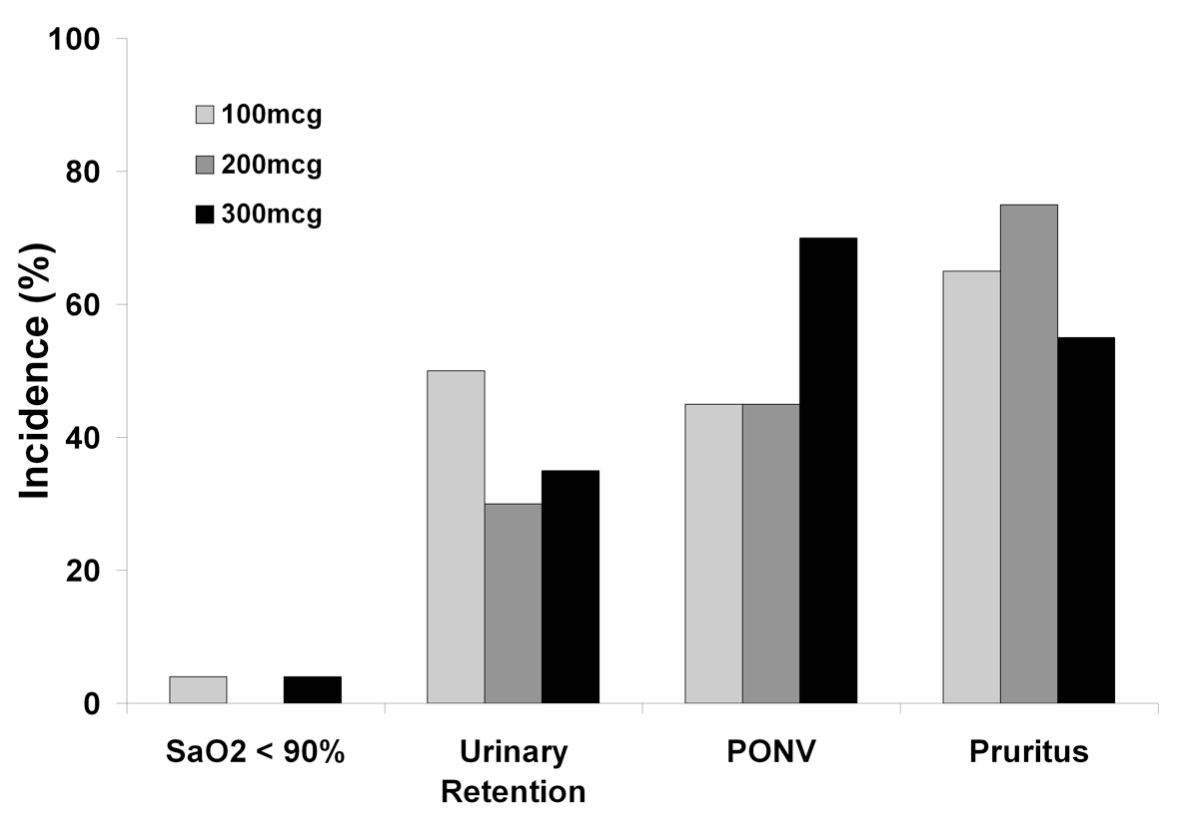
Incidence of side-effects, including arterial desaturation below 90%, urinary retention, postoperative nausea and vomiting (PONV) and pruritus in each group over the first 24 postoperative hours (P > 0.05, Chi Square, for all comparisons).

There was no evidence of significant respiratory depression at any of the doses of IT morphine studied. There was no difference in the number of episodes of reduced respiratory rate (respiratory rate < 12) in the first 24 postoperative hours in any group (Table 2). There was no difference in the incidence of episodes of mild or moderate hypoxemia between the three groups and all patients whose SpO_2 _fell below 94% responded to 40% O_2 _via facemask (Figure 3; Table 2). One patient, in the 300 μg group, developed transient severe hypoxemia which responded to an increase in inspired oxygen concentration.

There were no significant between group differences in the incidence or severity of PONV (Figure 3; Tables 2, 3). Specifically, there was no between group difference in: median PONV score at any time point (Table 3); time to first request for anti-emetic therapy (Table 2); number of patients that required anti-emetic therapy (Table 2); or overall incidence of PONV (Figure 3).

**Table 3:** Data regarding postoperative nausea, sedation and pruritus scores.

**Variable**	**100 μg IT Morphine**	**200 μg IT Morphine**	**300 μg IT Morphine**
Postoperative Nausea Scores			
0 hrs	0 (0, 0)	0 (0, 0)	0 (0, 0)
4 hrs	0 (0, 0.5)	0 (0, 0)	0 (0, 2.5)
8 hrs	0 (0, 0)	0 (0, 2)	0 (0, 2)
12 hrs	0 (0, 0)	0 (0, 0)	0 (0, 0)
16 hrs	0 (0, 0)	0 (0, 0)	0 (0, 0)
20 hrs	0 (0, 0)	0 (0, 0)	0 (0, 0)
24 hrs	0 (0, 0)	0 (0, 0)	0 (0, 0)
Postoperative Pruritus Scores			
0 hrs	0 (0, 0)	0 (0, 0)	0 (0, 0.5)
4 hrs	0 (0, 2)	2 (0, 2)	0 (0, 2)
8 hrs	0 (0, 2)	0 (0, 2)	0 (0, 0)
12 hrs	0 (0, 0)	0 (0, 0.5)	0 (0, 0)
16 hrs	0 (0, 0)	0 (0, 0)	0 (0, 0)
20 hrs	0 (0, 0)	0 (0, 0)	0 (0, 0)
24 hrs	0 (0, 0)	0 (0, 0)	0 (0, 2)
Postoperative Sedation Scores			
0 hrs	1 (1, 1)	1 (1, 1)	1 (1, 1)
4 hrs	1 (1, 2)	1 (1, 1)	1 (1, 3)
8 hrs	1 (1, 4)	1 (1, 1.25)	1 (1, 4)
12 hrs	4 (1, 4)	1 (1, 4)	4 (1, 4)
16 hrs	1 (1, 4)	1 (1, 4)	4 (1, 4)
20 hrs	1 (1, 1)	1 (1, 1)	1 (1, 1)
24 hrs	1 (1, 1)	1 (1, 1)	1 (1, 1)

**Notes**: Data are presented as median (interquartile range).

Abbreviations: IT, intrathecal.

There were no significant between group differences in the incidence or severity of pruritus (Figure 3; Tables 2, 3). Specifically, there was no between group difference in: median pruritus score at any time point (Table 3); number of patients that required anti-pruritic therapy (Table 2); or overall incidence of pruritus (Figure 3).

There were no between group differences in sedation scores (Table 3), or in the number of patients that developed significant sedation, which was defined as a sedation score of 5 (Table 2). There were no between group differences in the requirement for urethral catheterization (Figure 3).

## Discussion

This study demonstrates several important points. First, doses of 200 μg and 300 μg IT morphine provide comparable and effective postoperative analgesia in patients undergoing elective total knee arthroplasty. Second, 100 μg of IT morphine was not as effective in providing analgesia compared to the higher doses, and constitutes an inadequate dose of IT morphine in this patient group. Third, the incidence of respiratory depression and significant postoperative hypoxemia is low. No patient that received 200 μg of IT morphine developed severe arterial hypoxaemia in this study, attesting to the safety of this dose of IT morphine in this patient group. In summary, 200 μg IT morphine appears to provide comparable analgesia to 300 μg, and appears to be the best balance between analgesic efficacy and side-effect profile in this patient group.

Our study clearly demonstrates that both 200 μg and 300 μg of IT morphine provide comparable analgesic efficacy in patients undergoing elective knee arthroplasty. Both regimens provided excellent analgesia and with a low requirement for rescue analgesia in the first 24 hours postoperatively. There was no difference in postoperative VAS scores at any time point between patients receiving 200 μg and 300 μg of IT morphine. In addition, there was no difference in the duration of analgesia provided by these doses, or in the need for or amount of supplemental analgesic therapy required. We have demonstrated that 100 μg does not provide a comparable level of analgesia to that provided by 200 μg or 300 μg of IT morphine, suggesting that this constitutes an inadequate dose, and therefore should not be used in this patient group.

The finding that 300 μg is an effective dose of IT morphine in patients undergoing elective total knee arthroplasty has been demonstrated in previous studies [2]. We demonstrate for the first time that comparable levels of postoperative analgesia can be achieved with 200 μg of IT morphine. This finding contrasts to some extent with previous findings. Rathmell et al could not find evidence that doses of IT morphine up to 300 μg provided significant analgesia in this population [13]. However, this study included patients undergoing both hip and knee arthroplasty, and studied multiple doses of IT morphine. Consequently the numbers of patients in each group was relatively small, reducing study power. Bowrey et al did demonstrate that 500 μg intrathecal morphine produced better analgesia than 200 μg after knee replacement [1]. However, the risk of serious side effects, particularly respiratory depression is dose dependent [11,14]. Consequently, ASA guidelines advocate that the lowest efficacious dose of neuraxial opioids be used to minimize this risk [14].

Respiratory depression, the most serious side-effect of IT morphine, is more likely in the older patient, such as this patient group [15]. Volunteer studies have demonstrated that, while higher doses of IT morphine result in profound and prolonged late respiratory depression, doses as low as 200 μg may also produce significant respiratory depression [11]. In additional similar studies, 300 μg of IT morphine potently depressed the ventilatory response to hypoxia [16]. These data emphasize the need to determine the lowest effective dose of IT morphine in this patient population. In our study, one patient that received 300 μg IT morphine developed transient severe hypoxemia, which responded to administration of 60% Oxygen. None of the patients that received 100 μg or 200 μg IT morphine developed severe hypoxemia in this study. This confirms previous finding attesting to the safety of IT morphine in this dose range [2,17-19].

The incidence and severity of PONV is a major concern regarding the use of IT morphine [3,6]. In this study, there was no between group difference in the incidence or severity of PONV, or in the need for anti-emetic therapy, following the use of 100 – 300 μg of IT morphine. This suggests that PONV may be less problematic within this dose range, and is in agreement with our previous finding that 50 – 200 μg of IT morphine does not contribute to the incidence of PONV in the setting of elective hip arthroplasty under spinal anaesthesia [17].

Pruritus has been identified as a major side effect associated of IT opioids, and may contribute significantly to patient discomfort [3,6]. It has been proposed that this side effect is centrally mediated by μ-opioid receptors [20]. In this study, the incidence of pruritus was high in all groups, averaging 60% across the groups. There were no between group differences in the incidence or severity of pruritus, or in the need for anti-pruritic therapy, following the use of 100 – 300 μg of IT morphine. This contrasts with previous findings, from our group and others [7,17], that the incidence of pruritus following intrathecal morphine is dose dependent in patients following hip arthroplasty. Sedation did not appear to be a problem at the doses of IT morphine used in this study. The incidence of urinary retention did not appear to be related to the dose of IT morphine administered.

An important limitation in regard to our data for the side-effect profiles of the different doses of intrathecal morphine is that the study was powered to detect differences in analgesic efficacy, rather than in the incidence of side effects. Therefore, it remains possible that between group differences in more subtle, but nevertheless clinically important side effects, were not detected in this study. Caution is therefore warranted, particularly given the fact that doses of IT morphine as low as 200 μg has been demonstrated to produce significant respiratory depression in other studies [11]. These findings emphasize the need for appropriate monitoring of all patients receiving intrathecal morphine as part of their postoperative analgesic regimen.

## Conclusion

We conclude that administration of 200 μg provides equally effective post operative analgesia as 300 μg of IT morphine. Conversely, 100 μg of IT morphine provided less effective postoperative analgesia in these patients. We therefore recommend that 200 μg IT morphine be used for postoperative analgesia in patients undergoing knee arthroplasty.

## Abbreviations

ANOVA: analysis of variance; AUC: Area under the curve; HDU: High Dependency Unit; IT: Intrathecal; L3/4: Lumbar 3/4 intervertebral space; mg: milligrams; mL: mililitres; μg: micrograms; NaCl: Sodium Chloride; PONV: postoperative nausea and vomiting; SaO_2_: Arterial oxygen saturation; SEM: standard error of the mean; VAS: Visual analogue scale.

## Competing interests

The authors declare that they have no competing interests.

## Authors' contributions

PH conceived of the study, and participated in its design and execution and helped to draft the manuscript. BA, PG and BK participated in the study, recruited patients, and helped to draft the manuscript. JL participated in the design and coordination of the study, performed the statistical analysis, and helped to draft the manuscript. All authors read and approved the final manuscript.

## Pre-publication history

The pre-publication history for this paper can be accessed here:



## Supplementary Material

Additional file 1 Study Flowchart Hassett et al. This is a flow chart which provides full details of the patients screened, consented and enrolled in the study, and subjected to statistical analysis. Click here for file

## References

[B1] Bowrey S, Hamer J, Bowler I, Symonds C, Hall JE (2005). A comparison of 0.2 and 0.5 mg intrathecal morphine for postoperative analgesia after total knee replacement. Anaesthesia.

[B2] Cole PJ, Craske DA, Wheartley RG (2000). Efficacy and respiratory effects of low-dose spinal morphine for post-operative analgesia following knee arthroplasty. Br J Anaest.

[B3] Jacobson L, Chabal C, Brody MC (1988). A dose-response study of intrathecal morphine: efficacy, duration, optimal dose, and side effects. Anesth Analg.

[B4] Goodarzi M, Narasimhan RR (2001). The effect of Large-Dose Intrathecal Opioids on the Autonomic Nervous System. Anesth Analg.

[B5] Lydon AM, Cooke T, Duggan F, Shorten GD (1999). Delayed postoperative gastric emptying following intrathecal morphine and intrathecal bupivacaine. Can J Anaesth.

[B6] Carpenter RL, Caplan RA, Brown DL, Stephenson C, Wu R (1992). Incidence and risk factors for side effects of spinal anaesthesia. Anesthesiology.

[B7] Slappendel R, Weber EWG, Benraad B, van Limbeek J, Dirksen R (2000). Itching after intrathecal morphine. Incidence and treatment. Eur J Anaest.

[B8] Clergue F, Montembault C, Despierres O, Ghesquiere F, Harari A, Viars P (1984). Respiratory effects of intrathecal morphine after upper abdominal surgery. Anesthesiology.

[B9] Glass PS (1984). Respiratory depression following only 0.4 mg of intrathecal morphine. Anesthesiology.

[B10] Grattidge P (1998). Nausea and vomiting after major arthroplasty with spinal anaesthesia including morphine: a randomised trial of subhypnotic propofol infusion as prophylaxis. Acta anaesthesiologica Scandinavica.

[B11] Bailey PL, Rhondeau S, Schafer PG, Lu JK, Timmins BS, Foster W, Pace NL, Stanley TH (1993). Dose response pharmacology of intrathecal morphine in human volunteers. Anaesthesiology.

[B12] Laffey JG, Flynn N (2001). Low-dose spinal morphine for postoperative analgesia following knee arthroplasty. Br J Anaesth.

[B13] Rathmell JP, Pino CA, Taylor R, Patrin T, Viani BA (2003). Intrathecal morphine for postoperative analgesia: a randomized, controlled, dose-ranging study after hip and knee arthroplasty. Anesth Analg.

[B14] Horlocker TS (2007). Practice Guidelines for the Prevention, Detection and Management of Respiratory Depression Associated with Neuraxial Opioid Administration: Preliminary Report by ASA Task Force on Neuraxial Anesthesia. ASA Newsletter.

[B15] Gjessing J, Tomlin PJ (1981). Postoperative pain control with intrathecal morphine. Anaesthesia.

[B16] Bailey PL, Lu JK, Pace NL, Orr JA, White JL, Hamber EA, Slawson MH, Crouch DJ, Rollins DE (2000). Effects of intrathecal morphine on the ventilatory response to hypoxia. N Engl J Med.

[B17] Murphy PM, Stack D, Kinirons B, Laffey JG (2003). Optimizing the dose of intrathecal morphine in older patients undergoing hip arthroplasty. Anesth Analg.

[B18] Mendieta Sanchez JM, Fernandez-Liesa JI, Marco G, Panadero A, Sanchez-Ledesma MJ, Macias A (1999). Efficacy of 0.1 mg of subarachnoid morphine combined with bupivacaine on postoperative analgesia in total hip arthroplasty[Article in Spanish]. Rev Esp Anestesiol Reanim.

[B19] Slappendel R, Weber EW, Dirksen R, Gielen MJ, van Limbeek J (1999). Optimization of the dose of intrathecal morphine in total hip surgery: a dose-finding study. Anesth Analg.

[B20] Tohda C, Yamaguchi T, Kuraishi Y (1997). Intracisternal injection of opioids induces itch-associated response through mu-opioid receptors in mice. Jpn J Pharmacol.

