# Supplementary material for: Determination of the efficacy and side-effect profile of lower doses of intrathecal morphine in patients undergoing total knee arthroplasty
Source: BMC Anesthesiol. 2008 Sep 24;8:5. doi: 10.1186/1471-2253-8-5 (PMC2559822; doi:10.1186/1471-2253-8-5)
Supplement: Additional file 1 — Study Flowchart Hassett et al. This is a flow chart which provides full details of the patients screened, consented and enrolled in the study, and subjected to statistical analysis. [file 1471-2253-8-5-S1.doc]

# Study Flowchart

Assessed for Eligibility (n = 80)

Excluded (n = 20)

Not meeting inclusion criteria (n = 19)

15 deemed not suitable for spinal anaesthesia

4 history severe opioid induced nausea

Refused to participate (n = 1)

Other reasons (n = 0)

**Enrollment**

**100μg Morpine**

**300μg Morpine**

**Follow-Up**

**200μg Morpine**

**Analysis**

Analyzed (n = 20)

Excluded from analysis (n = 0)

Give reasons

Lost to follow-up (n = 0)

### Discontinued intervention

(n = 0)

#

### Allocated to intervention

(n = 20)

### Received allocated intervention

(n = 20)

# Did not receive allocated intervention

(n = 0)

Give reasons: N/A

Lost to follow-up (n = 0)

Discontinued intervention

(n = 0)

#

### Allocated to intervention

(n = 20)

### Received allocated intervention

(n = 20)

### Did not receive allocated intervention

(n = 0)

### Give reasons: N/A

Analyzed (n= 20)

Excluded from analysis (n = 0)

### Randomized

### Allocated to intervention

(n = 20)

### Received allocated intervention

(n = 20)

# Did not receive allocated intervention

(n = 0)

Give reasons: N/A

Lost to follow-up (n = 0)

### Discontinued intervention

(n = 0)

#

Analyzed (n = 20)

Excluded from analysis (n = 0)

Give reasons
